# Nosocomial *Acinetobacter baumannii* Infections and Changing Antibiotic Resistance

**DOI:** 10.12669/pjms.295.3885

**Published:** 2013

**Authors:** Ismail Necati Hakyemez, Abdulkadir Kucukbayrak, Tekin Tas, Aslihan Burcu Yikilgan, Akcan Akkaya, Aliye Yasayacak, Hayrettin Akdeniz

**Affiliations:** 1 Ismail Necati Hakyemez, Specialist of Infectious Diseases and Clinical Microbiology, Sevket Yilmaz Training and Research Hospital, Bursa, Turkey.; 2 Abdulkadir Kucukbayrak, Assistant Professor of Infectious Diseases and Clinical Microbiology, Abant Izzet Baysal University Medical Faculty, Bolu, Turkey.; 3 Tekin Tas, Assistant Professor of Medical Microbiology, Abant Izzet Baysal University Medical Faculty, Bolu, Turkey.; 4 Aslihan Burcu Yikilgan, Resident Physician of Infectious Diseases and Clinical Microbiology, Abant Izzet Baysal University Medical Faculty, Bolu, Turkey.; 5 Akcan Akkaya, Assistant Professor of Anesthesiology and Reanimation, Abant Izzet Baysal University Medical Faculty, Bolu, Turkey.; 6 Aliye Yasayacak, Nurse of Infectious Diseases and Clinical Microbiology, Abant Izzet Baysal University Medical Faculty, Bolu, Turkey.; 7 Hayrettin Akdeniz, Professor of Infectious Diseases and Clinical Microbiology, Abant Izzet Baysal University Medical Faculty, Bolu, Turkey.

**Keywords:** *Acinetobacter baumannii*, Antibiotic resistance, Nosocomial infections

## Abstract

***Objectives:*** In the intensive care setting,* Acinetobacter baumannii* causes ventilator-associated pneumonia and other nosocomial infections that are difficult to treat. Objective of this study was to investigate nosocomial *A. baumannii* infections and its changing antibiotic resistance.

***Methods:*** A total of 56 patients diagnosed with *A.baumannii* infections between January 2009 and December 2011 were included in the study. Diagnosis for nosocomial infections was established according to the CDC (Centers for Disease Control and Prevention) criteria. Identification of the agents isolated was carried out using conventional methods and VITEK 2 automated system, while antibiotic sensitivity testing was performed through VITEK 2 AST-N090 automated system.

***Results:*** The most common infection was nosocomial pneumonia by 43%, among which 46% were ventilator-associated pneumonia. Considering all years, the most effective antibiotics on the isolated strains were found as colistin, tigecycline, imipenem and meropenem. However resistance to imipenem and meropenem was observed to increase over years.

***Conclusion:*** The issue of increased resistance to antibiotics poses difficulty in treatment of *A. baumannii* infections which in turn increases the rate of mortality and cost. In order to prevent development of resistance, antibiotics must be used in an appropriate way in accompanied with proper guidance.

## INTRODUCTION

In the intensive setting,* Acinetobacter spp.,* increasingly causes nosocomial infections with mortality.^1^ In the clinical samples, the most commonly encountered opportunistic pathogen is *Acinetobacter baumannii* and because of its ability for colonization to the hospital setting and developing resistance, it leads to nosocomial infections that are difficult to treat.^2^ The most common and serious MDR pathogens take place in the abbreviation known as "ESKAPE" (*E. faecium, S. aureus, K. pneumoniae, A. baumannii, P. aeruginosa ve Enterobacter spp*.).^3^ *A. baumannii* colonizes in the respiratory tract, skin, urinary system and gastrointestinal system, and frequently leads to pneumonia, surgical site infections, central catheter-related blood circulatory infection, probe-related urinary system infections and rarely community acquired pneumonia, meningitis, mediastinitis, osteomyelitis and cholangitis.^4^ Immunosuppression, use of wide spectrum antibiotics, respiratory tract interventions and intravascular interventions are predisposing factors for development of infections.^5^

Because of the recently increasing resistance to carbapenems and studies reporting strains that are resistant to colistin, treatment is almost impossible in some cases. However, despite the developing resistance, combination of colistin and sulbactam seems to be the best treatment option in majority of the patients.^6^ Objective of this study was to evaluate changing antibiotics resistance in *A. baumannii* infections over years.

## METHODS

***Bacterial Isolates:*** In this study, the data of 56 in-patients diagnosed with nosocomial infection in which *A. Baumannii* was the agent according to CDC (Centers for Disease Control and Prevention) in Abant Izzet Baysal University Medical Faculty Hospital between January 2009 and December 2011 were retrospectively evaluated. Nosocomial isolate was defined as isolate grown from specimen that was sampled after 48 hours of hospitalization. *A. baumannii* strains which were considered as colonization were excluded from the study.

***Laboratory identification:*** Patients information were obtained from laboratory records. Clinical samples collected from the patients were cultivated on 5% defibrinated sheep blood Colombia agar, Eosin Methylene Blue agar and Chocolate agar, and incubated at 37°C for 24 hours. Identification of the isolated microorganisms was carried out using conventional method and VITEK 2 automated system (bioMerieux Inc, Mercy L’etoil, Fransa).

***Antimicrobial Susceptibility Testing:*** Antibiotic sensitivity testing was performed through VITEK 2 AST-N090 (bioMerieux Inc, Mercy L’etoil, Fransa) automated system for amikacin, amoxicillin-clavulanate, cefepime, ciprofloxacin, colistin, gentamicin, imipenem, meropenem, piperacillin-tazobactam, tetracycline, tigecycline and trimethoprim-sulfamethoxazole. Outcomes were interpreted according to CLSI (The Clinical and Laboratory Standards Institute) standards.^7^

***Ethical considerations:*** Ethical approval as obtained from the Ethical Committee of Faculty of Medicine, Abant Izzet Baysal University.

***Statistical Analysis:*** Data analysis was done on Statistical Package for Social Sciences (SPSS), version 13.0. Data presented in the form of frequency and percentage.

## RESULTS

Of the patients diagnosed with* A.baumannii* infection, 37 were males and 19 females with a mean age of 61.5 ± 19.1. Distribution of the samples in which *A.baumannii* strains were isolated is given in Table-I, while underlying diseases in the patients diagnosed with nosocomial infection and rate of device usage are shown in Table-II.

**Table-I:** Distribution of *A.baumannii* stratins according to the departments

*Departments*	*n (%)*
Reanimation ICU	27 (48.4)
Emergency Department ICU	4 (7.2)
Cardiovascular surgery ICU	1 (1.7)
Coronary ICU	1 (1.7)
Intensive care units	33 (59)
Cardiovascular surgery	8 (14.5)
Internal medicine	5 (8.9)
Urology	3 (5.5)
General surgery	2 (3.5)
Neurosurgery	2 (3.5)
Plastic surgery	1 (1.7)
Orthopedics and Traumatology	1 (1.7)
Neurology	1 (1.7)
Non-intensive care unit	23 (41)
Total units	56 (100)

**Table-II:** Underlying diseases and rate of device usage

*Underlying disease*	*n (%)*
Cardiac disease	26 (46.4)
Surgical operation	24 (42.9)
Diabetes mellitus	22 (39.2)
Cerebrovascular disease	16 (28.6)
Renal failure	14 (25)
Chronic obstructive pulmonary disease	11 (19.6)
Trauma	8 (14.2)
Device usage rates	n (%)
Urinary catheter	43 (76.8)
Central venous catheter	40 (71.4)
Mechanical ventilation	33 (58.9)

Forty-one (73.2%) patients were using wide spectrum antibiotic before the diagnosis. Mean duration between the hospitalization and diagnosis of infection was 23.1±21.5 days. Rate of mortality was 39.2% for all years, while this rate was found to be high as 46.1% in 2011. Distribution of nosocomial infections is presented in Table-III.

**Table-III:** Distribution of nosocomial infection types

*Nosocomial infection types*	*n (%)*
Ventilator-Associated pneumonia (VAP)	11 (19.6)
Nosocomial Pneumonia (NP); including VAP	24 (42.8)
Central Venous Catheter-Related Bloodstream Infection (CRBSI)	11 (19.6)
Surgical Site Infection (SSI)	11 (19.6)
Catheter-Associated Urinary Tract Infection (CAUTI)	8 (14.2)
Mediastinitis	1 (1.8)
Soft tissue infection	1 (1.8)

Evaluating all years, the most effective antibiotics on *A.baumannii* strains were found as colistin, tigecycline, imipenem and meropenem. However, a significant increase in antibiotic resistance against imipenem and meropenem was found in 2011. Colistin and tigecycline were studied only in 2011 and no resistant strain was found (Table-IV). 

**Table-IV:** Resistance rates of nosocomial *A. baumannii* strains between 2009 and 2011

*Antibiotics*	*2009 (n:14)*	*2010 (n:16)*	*2011 (n:26)*	*2009-2011 * *(n:56)*
Amikacin	12 (83.3)	16 (93.7)	16 (81.2)	44 (86.3)
Amoxicillin-clavulanate	14 (85.7)	7 (100)	19 (89.4)	40 (90)
Cefepime	14 (100)	11 (100)	20 (90)	45 (95.5)
Ciprofloxacin	14 (85.7)	15 (86.6)	24 (83.3)	53 (84.9)
Colistin	--------	---------	16 (0)	16 (0)
Gentamicin	14 (85.7)	15 (60)	18 (83.3)	47 (76.5)
Imipenem	10 (0)	16 (12.5)	26 (88.4)	52 (48.1)
Meropenem	10 (20)	6 (0)	16 (93.7)	32 (53.1)
Piperacillin-tazobactam	10 (80)	7 (100)	15 (93.3)	28 (89.2)
Tetracycline	13 (23.1)	15 (40)	18 (72)	53 (50.9)
Tigecycline	---------	---------	13 (0)	13 (0)
Trimethoprim-sulfamethoxazole	14 (78.5)	16 (93.7)	25 (76)	55 (81.8)

## DISCUSSION

Multidrug resistant (MDR) *A. baumannii* is an opportunistic pathogen developing especially in the intensive care settings leading to infections such as bacteremia, Nosocomial Pneumonia, VAP, meningitis, CAUTI, central venous CRBSI and wound infection. Incidence of *A. baumannii* infections have increased in a number of regions in the world in the last decade and have caused to epidemics depending on the ability of this organism. In general, antibiotics effective against *A. baumannii* infections are carbapenems, polymyxins, sulbactam, tigecycline, and aminoglycosides.^8^^,^^9^

*A. baumannii* is the most commonly isolated from the respiratory tract, blood culture, wound and urine samples.^10^ That patients hospitalized in ICU mostly received wide spectrum antibiotic treatment leads to isolation of *A.baumannii* strains frequently from these units.^11^ It is reported to be the most commonly isolated agent in Reanimation ICU.^12^ In this study, 59% of *A.baumannii *strains were isolated from ICU (Reanimation 48%).

In the antibiotic sensitivity studies conducted in our country, resistance rates of* A.baumannii* are reported as 32-100% against ciprofloxacin, 91-100% against cefepime, 90-92% against piperacillin-tazobactam, 24-94% against amikacin and 18-85% against gentamicin.^13^ In this study, we found the resistance rates as 84.9 against ciprofloxacin, 95.5% against cefepime, 89.2% against piperacillin-tazobactam, 86.3% against amikacin and 76.5% against gentamicin and these results will remove these drugs from being an option for treatment.

The most important problem in treatment of *A.baumannii* infections is the increase of isolated strains resistant against multiple drugs together with the narrowing of the options in antibotics to be used in the treatment. Carbapenem resistance rates are increasing to such an extent to threaten the world and this situation is gradually becoming a routine phenotype for the microorganism. Therefore, in order to take the microorganism under control, infection control strategies must be focused on besides the treatment options.^14^ Increasing carbapenem resistance in the *A. baumannii* isolates has resulted from expansion of certain carbapenem-resistant clones.^15^ Carbapenems (imipenem, meropenem) remain valuable among the treatment options in combination therapies in the MDR strains. In 2011 report by National Hospital Infections Surveillance Network, carbapenem resistance was reported as 57-83% in *A.baumannii *strains isolated in the hospital infections.^16^ Iraz et al reported a high rate of carbapenem resistance by 92%.^12^ In our study, rates of resistance to carbapenems had been 0% for imipenem and 20% for meropenem in 2009, while these rates raised to very high values with 88.4% for imipenem and 93.7% for meropenem in 2011.

Increase of carbapenem resistance raises the fact that the re-use of old antibiotics like polymyxin B. Colistin has a lower rate of mortality than carbapenems in treatment of MDR infections. It is recommended to be combined with rifampicin, sulbactam and carbapenems.^17^ However, recently colistin resistance is reported worldwide, especially in Europe.^18^ In our country, a resistance has not yet been seen at a high level. Ergin et al. reported colistin resistance as 2% in *A.baumannii*. In our study, all the 13 strains were found to be colistin sensitive.^19^

Tigecycline is a glycylcycline derived antibiotic which is in vitro effective on MDR *A.baumannii*. Baadani et al^20^ found tigecycline resistance as 4.7-20.5% in the *A. baumannii* isolates in two different hospitals. Studies conducted in our country reported tigecycline resistance as 0-12%.^13^ No tigecycline-resistant strain was detected in our study.

Inappropriate treatment administrations directly affect mortality.^21^ In this study, rate of mortality was found as 39.2% for all years. In the conducted studies, rate of mortality in *A. baumannii* infections is reported between 22-44%.^22^ Lee et al demonstrated that in serious cases having *A. baumannii* bacteremia, 14-day mortality decreased to 13% from 29% with a proper antimicrobial treatment.^23^

It is inevitable for clinicians to develop a road map about the approaches to resistant *Acinetobacter* infections, including trainings on the infection control and proper antibiotic use based on the antibiotic sensitivity status in their regions. We believe that this approach will reduce the rates of resistant infections.
